# The CDC antimicrobial use measure is not ready for public reporting or value-based programs

**DOI:** 10.1017/ash.2023.143

**Published:** 2023-04-19

**Authors:** Nathan R. Shively, Daniel J. Morgan

**Affiliations:** 1 Division of Infectious Diseases, Allegheny Health Network, Pittsburgh, Pennsylvania; 2 Department of Epidemiology and Public Health, University of Maryland School of Medicine, Baltimore, Maryland; 3 Veterans’ Affairs (VA) Maryland Healthcare System, Baltimore, Maryland

## Abstract

The standardized antimicrobial administration ratio (SAAR) is the metric for reporting antimicrobial use that hospitals will be mandated to use in 2024. We highlight limitations of the SAAR and caution against efforts to use it for public reporting and financial reimbursement. Before the SAAR is ready for public reporting, it needs to include patient-level risk adjustment and antimicrobial resistance data as well as improved hospital location options and revised antimicrobial agent groupings to appropriately reflect and incentivize important stewardship work.

Antimicrobial use (AU) tracking is a critical component of any antimicrobial stewardship program (ASP).^1^ Initially developed in 2015 by the Centers for Disease Control and Prevention (CDC), the standardized antimicrobial administration ratio (SAAR) is an AU measure that has potential to allow for national benchmarking and interfacility comparison.^2^ Thousands of hospitals have voluntarily submitted data to the National Healthcare Safety Network (NHSN) Antimicrobial Use and Resistance (AUR) Option,^2^ and we believe that the SAAR has significant promise as a public health and stewardship tool. However, the Centers for Medicare and Medicaid Services (CMS) has announced that beginning in 2024, all acute-care hospitals and critical-access hospitals will be required to report to the NHSN AUR module.^3^ In light of this change, our commentary is meant to caution against public reporting and inclusion in value-based programs tied to reimbursement. Rushing the measure to have consequences for hospital reimbursement before it is ready risks unintended consequences, including a forced redistribution of limited resources, and may be harmful to hospitals and ultimately, patient care. We critique the SAAR and offer past examples of similarly flawed measures used for public reporting without an adequate evidence base.

## The SAAR

The SAAR is similar to the more well-established standardized infection ratio (SIR) used to track healthcare-associated infections (HAIs). Similarities include the use of an observed-to-predicted framework to guide facilities. However, a critical difference is that while the optimal number of HAIs is zero, the optimal amount of antibiotic use is unknown. Antibiotics are lifesaving medications to patients with severe infections. The ideal amount of antibiotic use is an elusive benchmark because it can be variable by setting and patient and is, to some degree, subjective.^4^ Most inpatient ASPs determine appropriate use through some amount of chart audit and feedback, which allows for incorporation of individual clinical scenarios and patient-level variables in decision making. Developing a risk-adjusted SAAR for national benchmarking is a laudable goal, but for ASPs to be able to use it constructively, the details matter.

SAAR is not a single metric but a multifaceted one. The SAAR creates a metric for adult, pediatric, and neonatal populations using 17 patient location types across 22 antimicrobial agent categories. This framework results in 47 possible SAARs (Appendix 1).^5^ Certainly, variability in antimicrobial utilization would be expected between these types of units. However, a vast difference of expected use may remain even within these unit types, limiting the value in comparison. For example, a “hematology oncology” unit that cares primarily for solid-tumor cancer patients would have significantly different expected antibiotic use from one that cares primarily for hematologic malignancies with stem-cell transplant patients, where antimicrobial prophylaxis may be appropriate and where neutropenic fever is common. Similarly, in some facilities, solid-organ transplant units may be categorized as any of the ward types, and expected antimicrobial use would undoubtedly differ from similarly categorized wards that do not care for such patients. Although a facility stewardship program with specific knowledge of each of their units may be able to use the SAAR for internal benchmarking and initiatives, the absence of this level of detail would make broader comparisons at the regional, state, or national level of limited value. More importantly, a move toward pay for performance with financial penalties for higher-than-expected use, without such adjustments, would be a disservice to those facilities caring for more vulnerable patients and may increase disparities by income and race. Stewardship in immunocompromised patients is an important and growing area of focus,^6^ and peer comparison with similar units or facilities may be valuable for those caring for such patients. However, the SAAR requires further refinement to allow for such comparisons.^7^

Beyond the lack of granularity of location types, the current SAAR antimicrobial groupings are similarly too broad to allow for meaningful tracking or comparison. For example, ertapenem and fluoroquinolones are grouped with cefuroxime and ceftriaxone under “adult broad-spectrum antibacterial agents predominantly used for community-acquired infections.” Important stewardship work for community-acquired intra-abdominal infections may include promotion of second- or third-generation cephalosporin use plus metronidazole and discouragement of fluoroquinolone use (due to higher resistance expected for the typical pathogens and the adverse effect profile) and of ertapenem use (given lack of need for need for such broad coverage in most locations). However, such work would be invisible within the current SAAR categorization. Worse, de-escalation from ertapenem would actually increase the “all antibacterial agents” SAAR. Under threat of financial penalties, it is not difficult to imagine a facility moving from ceftriaxone and metronidazole to ertapenem for such infections, which would improve their SAAR but would be worse for patient care, selection of resistance, and overall cost of care. There are similar potential issues in other SAAR antimicrobial groupings (Table 1).

**Table 1.** Selected Standardized Antimicrobial Administration Ratio (SAAR) Antimicrobial Groupings and Potential Issues

SAAR Antimicrobial Agent Category	Included Antimicrobials (Grouped by Category)			Potential Issues
Adult Broad-spectrum antibacterial agents predominantly used for hospital-onset infections	Cefepime Ceftazidime Piperacillin/tazobactam	Aztreonam (IV only) Amikacin (IV only) Gentamicin (IV only) Tobramycin (IV only)	Doripenem Imipenem/cilastatin Meropenem	• Carbapenems, aztreonam, and potentially aminoglycosides often restricted more than third- and fourth-generation cephalosporins or piperacillin-tazobactam. • Important de-escalation stewardship work occurs within this category that is invisible with this grouping.
Adult Broad-spectrum antibacterial agents predominantly used for community-acquired infections	Cefaclor Cefdinir Cefixime Cefotaxime Cefpodoxime Cefprozil Ceftriaxone Cefuroxime	Ciprofloxacin Gemifloxacin Levofloxacin Moxifloxacin	Ertapenem	• The differences in spectrum and adverse effect profile of included agents lead to important stewardship work within this category (see text).
Adult Antibacterial agents predominantly used for resistant gram-positive infections	Vancomycin (IV only)	Dalbavancin Oritavancin Ceftaroline Telavancin	Daptomycin Linezolid Tedizolid Quinupristin/Dalfopristin	• Dalbavancin and oritavancin would allow hospitals to minimize this SAAR substantially. • Limited use of VRE active agents over vancomycin would not be visible. • De-escalation to vancomycin from other agents in this category would not be trackable.
Adult Antifungal agents predominantly used for invasive candidiasis	Fluconazole	Anidulafungin Caspofungin Micafungin		• De-escalation from micafungin or similar to fluconazole would not be visible.

Note. SAAR, standardized antimicrobial administration ratio; IV, intravenous; VRE, vancomycin-resistant enterococci.

To further demonstrate the need for risk adjustment, consider a region in which the community rate of ESBL *Enterobacterales* is high enough to warrant empiric ertapenem for community-acquired intra-abdominal infections. Even if antimicrobial grouping is rectified in future SAAR iterations, to appropriately compare facilities regarding expected antimicrobial utilization, antimicrobial resistance data would need to be considered. This analysis may be possible with the requirement of submission to the Antimicrobial Resistance Option, and preliminary investigations into utilization of antimicrobial resistance data have been done.^8^ However, any pay-for-performance initiative designed prior to its inclusion would be premature.

Importantly, the SAAR does not adjust for any patient-level factors. Patient-level data are needed to appropriately benchmark antimicrobial utilization.^9^ Because inclusion of patient-level variables can substantially influence how hospitals rank,^10^ further research into which patient-level variables should be included is necessary to guide further refinement of the SAAR before any consideration of public reporting or using such data for reimbursement. Such refinement is being considered by the CDC.^2^

Finally, feasibility of mandated reporting must also be considered. Smaller-to-midsize community facilities often have fewer financial resources, lower content expertise, and less information technology capability to facilitate the required reporting. Lack of time, technical support, and salary support are known barriers for AUR reporting.^11^ Critical-access hospitals, which voluntarily report to the AU option at a lower percentage compared to all hospitals,^2^ will be required to report to NHSN by 2024.^3^ Although the CMS has delayed implementation of AUR reporting by a year from the previously proposed 2023, they estimate a median cost of $187,400 to purchase or build an AUR reporting solution.^3^ Especially for smaller facilities who do not already have this in place, mandating reporting by 2024 remains an aggressive timeline with considerable financial and administrative barriers.

### The slippery slope

Premature requirement of infectious disease metric reporting has occurred in the past, with negative impacts on patients. In 2004, The Joint Commission instituted a core measure for community-acquired pneumonia that included requirements for drawing blood cultures as well as administration of antibiotics within 4 hours of arrival at the emergency department.^12^ These measures, which were publicly reported and linked to reimbursement, led to unnecessary blood-culture ordering (the routine use of which may lead to false-positive results, unnecessary antibiotics, and increased length of stay^13^) that continued despite revisions to the measure to limit use to a sicker subset of patients.^14^ They also led to unnecessary antibiotic administration by emergency physicians to meet the metric.^15^ Not supported by high-quality evidence,^16^ the pneumonia measure has since been retired.

Similarly, adherence to the Severe Sepsis and Septic Shock Early Management Bundle (SEP-1), implemented by the CMS in 2015, has not led to improved patient outcomes,^17,18^ but these data continue to be required and publicly reported. Citing concerns of antibiotic overuse, the Infectious Diseases Society of America, with the support of the American College of Emergency Physicians, American Hospital Association, Pediatric Infectious Diseases Society, Society for Healthcare Epidemiology of America, Society of Hospital Medicine, and Society of Infectious Diseases Pharmacists, has called for major revisions to the measure.^19^ Although well intended, these examples should serve as warnings that national requirements that precede a firm evidence base can lead to wasteful or harmful patient care and unnecessary administrative burden. Mandated reporting of AU data to the NHSN is one step closer to mandated public reporting and use of the SAAR for financial penalties to hospitals, for which it is not nearly ready.

In summary, we applaud the CDC for continued exploration of the SAAR as a risk-adjusted, validated AU metric available for national benchmarking. However, the SAAR is not ready for mandatory use. It needs granularity and risk adjustment for the metric to improve antibiotic use. The CDC is aware of many of these limitations to the SAAR and has highlighted the potential for improvements in future iterations.^2^ With AUR reporting now mandated by the CMS by 2024, we caution policy makers not to go further by requiring public reporting or inclusion of SAAR metrics in value-based programs because doing so prematurely could lead to significant unintended financial and, more importantly, patient safety consequences.

## Supplementary material

For supplementary material accompanying this paper visit http://doi.org/10.1017/ash.2023.143. click here to view supplementary material
