# Supplementary material for: The CDC antimicrobial use measure is not ready for public reporting or value-based programs
Source: Antimicrob Steward Healthc Epidemiol. 2023 Apr 19;3(1):e77. doi: 10.1017/ash.2023.143 (PMC10127230; doi:10.1017/ash.2023.143)
Supplement: Supplementary file 1 [file S2732494X23001432sup001.docx]

Appendix 1. Standardized Antimicrobial Administration Ratio (SAAR) Types

| **SAAR Antimicrobial Agent Category** | **Locations (Adult and Pediatric)^a^** | **Adult** | **Pediatric** | **Neonatal** |
| --- | --- | --- | --- | --- |
| All Antibacterial Agents | All eligible SAAR locations | X | X | X |
| Broad spectrum antibacterial agents predominantly used for hospital-onset infections | Medical, Medical-Surgical, Surgical ICUs^b^ | X | X | X |
|  | Medical, Medical-Surgical, Surgical Wards | X | X |  |
|  | Step Down Units | X |  |  |
|  | General Hematology-Oncology Wards | X |  |  |
| Broad spectrum antibacterial agents predominantly used for community-acquired infections | Medical, Medical-Surgical, Surgical ICUs^b^ | X | X |  |
|  | Medical, Medical-Surgical, Surgical Wards | X | X |  |
|  | Step Down Units | X |  |  |
|  | General Hematology-Oncology Wards | X |  |  |
| Antibacterial agents predominantly used for resistant Gram-positive infections | Medical, Medical-Surgical, Surgical ICUs^b^ | X | X |  |
|  | Medical, Medical-Surgical, Surgical Wards | X | X |  |
|  | Step Down Units | X |  |  |
|  | General Hematology-Oncology Wards | X |  |  |
| Narrow spectrum beta-lactam agents | Medical, Medical-Surgical, Surgical ICUs^b^ | X | X |  |
|  | Medical, Medical-Surgical, Surgical Wards | X | X |  |
|  | Step Down Units | X |  |  |
|  | General Hematology-Oncology Wards | X |  |  |
| Antibacterial agents posing the highest risk for *Clostridioides difficile* infection | Medical, Medical-Surgical, Surgical ICUs^b^ | X | X |  |
|  | Medical, Medical-Surgical, Surgical Wards | X | X |  |
|  | Step Down Units | X |  |  |
|  | General Hematology-Oncology Wards | X |  |  |
| Antifungal agents predominantly used for invasive candidiasis | Medical, Medical-Surgical, Surgical ICUs^b^ | X | X |  |
|  | Medical, Medical-Surgical, Surgical Wards | X | X |  |
|  | Step Down Units | X |  |  |
|  | General Hematology-Oncology Wards | X |  |  |
| Azithromycin | Medical and Medical-Surgical ICUs |  | X |  |
|  | Medical, Medical-Surgical, Surgical Wards |  | X |  |
| Vancomycin predominantly used for treatment of late-onset sepsis |  |  |  | X |
| Third generation Cephalosporins |  |  |  | X |
| Ampicillin predominantly used for treatment of early-onset sepsis |  |  |  | X |
| Aminoglycosides predominantly used for treatment of early-onset and late-onset sepsis |  |  |  | X |
| Fluconazole predominantly used for candidiasis |  |  |  | X |

Note. ICU, intensive care unit.
^a^Neonatal Locations are Special Care Nursery (Level II), Neonatal Critical Care (Level II/III), Neonatal Critical Care (Level III), and Neonatal Critical Care (Level IV)
^b^Medical and Medical-surgical ICUs only for Pediatrics
